# Stability of Filled PDMS Pervaporation Membranes in Bio-Ethanol Recovery from a Real Fermentation Broth

**DOI:** 10.3390/membranes13110863

**Published:** 2023-10-27

**Authors:** Cédric Van Goethem, Parimal V. Naik, Miet Van de Velde, Jim Van Durme, Alex Verplaetse, Ivo F. J. Vankelecom

**Affiliations:** 1 Membrane Technology Group, Centre for Membrane Separations, Adsorption, Catalysis and Spectroscopy for Sustainable Solutions (cMACS), Department of Microbial and Molecular Systems, KU Leuven, Celestijnenlaan 200F, 3001 Leuven, Belgium; 2 Laboratory of Enzyme, Fermentation and Brewery Technology, Cluster for Bioengineering Technology, Department of Microbial and Molecular Systems, KU Leuven, Gebroeders De Smetstraat 1, 9000 Ghent, Belgium; 3 Research Group Molecular Odor Chemistry, KU Leuven Technology Campus Ghent, Gebroeders De Smetstraat 1, 9000 Ghent, Belgium

**Keywords:** pervaporation, bio-ethanol, metal–organic framework, stability, PDMS, MMM

## Abstract

Mixed matrix membranes (MMMs) have shown great potential in pervaporation (PV). As for many novel membrane materials however, lab-scale testing often involves synthetic feed solutions composed of mixed pure components, overlooking the possibly complex interactions and effects caused by the numerous other components in a real PV feed. This work studies the performance of MMMs with two different types of fillers, a core-shell material consisting of ZIF-8 coated on mesoporous silica and a hollow sphere of silicalite-1, in the PV of a real fermented wheat/hay straw hydrolysate broth for the production of bio-ethanol. All membranes, including a reference unfilled PDMS, show a declining permeability over time. Interestingly, the unfilled PDMS membrane maintains a stable separation factor, whereas the filled PDMS membranes rapidly lose selectivity to levels below that of the reference PDMS membrane. A membrane autopsy using XRD and SEM-EDX revealed an almost complete degradation of the crystalline ZIF-8 in the MMMs. Reference experiments with ZIF-8 nanoparticles in the fermentation broth demonstrated the influence of the broth on the ZIF-8 particles. However, the observed effects from the membrane autopsy could not exactly be replicated, likely due to distinct differences in conditions between the in-situ pervaporation process and the ex-situ reference experiments. These findings raise significant questions regarding the potential applicability of MOF-filled MMMs in real-feed pervaporation processes and, potentially, in harsh condition membrane separations in general. This study clearly confirms the importance of testing membranes in realistic conditions.

## 1. Introduction

Bio-based fuels have gained attention due to increasing concerns about CO_2_ emissions and the associated climate change. Ethanol production from so-called second generation biomass, such as switchgrass, corn stover, wheat or rice straw, via fermentation processes is considered to be a potential sustainable source of alternative liquid fuels [1,2,3]. Separations are an integral part of biofuel production through fermentation because the produced bio-ethanol needs to be dehumidified for it to be used as fuel, but more importantly, also because the fermentation process suffers from severe product inhibition due to the constant increase in concentration of organic components [4]. As product inhibition significantly hampers the overall process performance, it is widely accepted that integrating fermentation processes with a suitable in-situ product recovery technique (e.g., gas stripping, liquid–liquid extraction or pervaporation) could help to overcome this problem [5]. Among them, pervaporation (PV) is regarded as one of the most promising techniques for the recovery of alcohols from fermentation broths because of its operation under mild conditions, and its energy and cost effectiveness [6,7]. PV is especially beneficial for the removal of minor components from a mixture as the heat of vaporization only has to be provided for the fraction of the feed that actually permeates the membrane. The separation in PV processes depends on the sorption of compounds into, and diffusion through the membrane [8]. As such, PV performance is strongly influenced by the membrane material, the process conditions (e.g., feed concentration and temperature), the specific interactions of the membrane with certain feed components, etc. [6]. Recovery of organic compounds from aqueous solutions (e.g., a fermentation broth) is typically achieved using hydrophobic membranes. Polydimethylsiloxane (PDMS) is one of the most commonly used materials for organophilic PV because of its easy manufacturing, cost effectiveness and chemical and thermal stability [7,9].

Compared to polymeric membranes, inorganic PV membranes often have a better chemical/thermal stability and combine this with a higher separation factor and flux. However, they suffer from brittleness and high price [6,10]. The combination of inorganic materials in a polymeric matrix to form so-called mixed matrix membranes (MMMs) offers the potential to improve the separation performance of polymeric membranes while not suffering from the issues associated with purely inorganic membranes [7,11,12]. A large variety of filler materials has been explored in MMMs for PV, ranging from zeolites [13,14] and oxides [15] to metal–organic frameworks (MOFs) [16,17,18]. MOFs in particular have received a lot of attention over the past years. MOFs are crystalline, porous structures formed from the combination of metal ions or clusters and organic linker molecules [19]. As such, a huge variety in structures and chemical functionality is possible [20], which is also reflected in the wide range of potential applications such as catalysis or adsorption [21,22]. Also within PV, MOFs can be diversely deployed. Hydrophobic structures such as ZIF-8 or ZIF-71 are ideal materials for organophilic PV [16,17,18,23,24,25], whereas more hydrophilic structures, e.g., UiO-66 or MOF-303, function optimally in dehydration processes [26,27].

One of the major hurdles in bridging novel PV membranes to industrial reality is the lack of membrane testing in realistic conditions. While this is in general true for membranes developed for any membrane process, also novel PV membranes are mostly tested only in synthetic feeds made of mixed pure components. Such synthetic feeds are easily available, easier to analyze and eliminate complex interactions between minor feed components and the membrane. While testing performance in synthetic feeds is absolutely necessary to screen novel materials or gain fundamental insight into membrane and transport properties, it is specifically the performance in complex realistic feed streams that will ultimately determine the potential for industrial applicability. Generally, the effects of complex feeds can be grouped into four categories. (i) First, there are the changes in feed properties due to complex intramolecular interactions in complex mixtures. The presence of salts and sugars in real feeds are prime examples thereof as their interactions with water molecules (electrostatic interactions in hydration shells with ions or hydrogen bonding for organic molecules) are known to decrease water activity while increasing the activity of organic components, often referred to as ‘salting-out’. This is known to lead to an increased flux of the organic component while lowering the water flux [28,29]. Lipnizki et al. observed an increase in propanol permeance and propanol to water separation factor in the presence of salts such as NaCl or MgCl_2_ [29]. (ii) Additionally, all feed components will interact with, and partition into the membrane, leading to competitive sorption with the main targeted molecule. An important distinction has to be made between permeating components and low vapor pressure components that could, in practice, permanently occupy sorption sites. Succinic acid and glycerol, with boiling points around 235 and 290 °C, could be considered examples of the latter and have indeed been associated with flux decline [30]. (iii) In all aforementioned considerations, the membrane is assumed to remain unaffected. Degradation of the membrane material (filler as well as polymer in the case of MMMs) can however not be excluded, especially with real feeds. Many components present in a real feed might lead to polymer or filler degradation. Bowen et al. suggested the irreversible flux decline they observed for an acetic and succinic acid-containing feed might, amongst other reasons, be due to Al leaching from the ZSM-5 filler or reaction with the polymer [13]. Such complex effects of filler degradation have gained more attention in past years for all types of MMMs [31]. (iv) Finally, process operating conditions are also crucial information herein, as the effect of a certain component can vary drastically with parameters such as, e.g., pH. Bowen et al. showed that the effect of organic acids, such as acetic and succinic acid, depends on their ionization and thus on pH [13]. When the carboxylate species dominates the protonated carboxylic acid form, little effect was observed because of negligible partitioning into the membrane, whereas at lower pH, these components start to have important effects on overall PV performance. These are just general principles. Contradicting observations for the effect of the same component have indeed sometimes been made, highlighting the importance of studying realistic complex feeds for novel membranes. Glycerol is an example thereof, where often drastic decreases in flux are observed [30], while sometimes no significant effect on PV performance is observed [32].

MOF-filled MMMs for PV have so far not yet been tested in real feed streams. ZIF-8 is often considered a prototypical MOF because it is easy to prepare and requires only relatively cheap reagents. Its hydrophobic nature (mainly due to the methyl-group in the 2-methylimidazole linker) renders it ideal for organophilic PV. In previous work, ZIF-8 was grown on mesoporous silica spheres to guarantee fast mass transfer [33]. Hollow silicalite-1 spheres were developed for the same reason [34]. In this work, PDMS-based MMMs with these fillers (further denoted as ZIF-8 and silicalite-1 for ease of reading) were applied in the recovery of ethanol from a fermented wheat/hay straw hydrolysate broth. The PV performance of the MMMs and a reference unfilled PDMS membrane followed in time. A thorough membrane autopsy was performed on the ZIF-8 filled PDMS membrane after PV to analyze the effect of the fermented hydrolysate broth on the membrane and its filler. This work thus aims at contributing to the assessment of the technical feasibility of zeolite and MOF-filled MMMs in industrial bio-refineries.

## 2. Materials and Methods

### 2.1. Materials

PDMS (RTV-615, comprising two components A and B, chemical structure shown in Figure S1) was obtained from GE silicones (Brussels, Belgium), ethanol (99.9%) and toluene (99%) were purchased from VWR. Zn(NO_3_)_2_.6H_2_O and 2-methylimidazole (HMim) were purchased from Sigma Aldrich (Overijse, Belgium). Dimethylformamide (DMF) was obtained from Across Organics.

### 2.2. Membrane Preparation

Two PDMS-based MMMs were selected based on previous work: a 20 wt% ZIF-8 coated mesoporous silica sphere-filled PDMS membrane [33] and a 30 wt% hollow silicalite-1 sphere-filled PDMS membrane [34]. The membranes will be further denoted respectively as ZIF-8 and silicalite-1 filled membranes for the ease of reading. Thick, dense membranes were used such that a sufficient amount of filler membrane is present in the membranes to facilitate physicochemical characterization. The reader is referred to the previous work for the experimental details on the filler preparation [33,34]. PDMS-based membranes were prepared from separate solutions of the PDMS-prepolymer and crosslinker in hexane. The filler particles were dispersed in hexane through 1 h of sonication. The three solutions were then mixed and stirred for 4 h at 60 °C to initiate the PDMS crosslinking. The resulting solution was then poured into a glass petri-dish and covered with a funnel to allow slow solvent evaporation. Final crosslinking of the membrane was completed through a 1 h heat treatment at 110 °C.

### 2.3. Hydrolysate Fermentation

Fermented hydrolysate broth (2 L) was prepared and supplied by the Enzyme, Fermentation and Brewing Technology center, KU Leuven Technology campus Ghent. Initially, yeast was cultivated in XP-medium containing 20 g/L xylose, 10 g/L yeast extract and 20 g/L bacteriological peptone. Hydrolysate prepared from 50% hay and 50% wheat straw was added on the second day. The hydrolysate was prepared by performing an alkaline pre-treatment with 0.4 M NaOH followed by an enzymatic hydrolysis with Cellic Ctec 2 (Novozymes). The hydrolysate was mixed before being added to the fermentor and pH was adjusted with NaOH during the fermentation. Final alcohol concentration in the hydrolysate was 1.3%. Additional ethanol was added to the fermentation broth to adjust the alcohol concentration to 6% to facilitate comparison with previous work.

### 2.4. Membrane Testing

PV experiments were carried out in a cross-flow pervaporation setup consisting of 3 cells in series, each with an active membrane area of 0.001589 m^2^ as shown in Figure S2. Cross-flow velocity was set to 1 L.min^−1^ to minimize concentration polarization, and the feed temperature was kept at 40 °C. A vacuum of <1 mbar was kept at the permeate side to provide a driving force for the transport across the membrane. Permeate was collected through a cold trap and analyzed through High Performance Liquid Chromatography (HPLC) with Refractive Index (RI) detection. The HPLC was performed with a Shodex (Resonac Europe, Germany) sugar SH1011 column which was eluted at 60 °C with 0.005 M H_2_SO_4_ at a flow rate of 0.6 mL.min^−1^.

### 2.5. Membrane Characterization

XRD patterns of the membranes were recorded in transmission mode on a STOE stadi P high-throughput X-ray diffractometer using CuKα radiation (1.5418 Å wavelength). Cross-section SEM images of the membranes were acquired using a JEOL (Tokyo, Japan) JSM-6010LV microscope operated at 10 kV. The membranes were fractured in liquid nitrogen, fixed on carbon tape and coated with a Au/Pd layer (JEOL Auto Fine Coater JFC-1300) prior to imaging. SEM-EDX experiments were performed using a Phillips/FEI (Eindhoven, The Netherlands) XL-30 SEM equipped with an FEG, operated at 10 kV. Samples were prepared as described above. ATR-FTIR spectra of the membranes were measured in ATR-mode on a Varian (Palo Alto, CA, USA) 670 FTIR spectrometer coupled to a Varian 620 IR microscope equipped with an ATR crystal.

### 2.6. ZIF-8 Stability in Fermented Hydrolysate

ZIF-8 particles with ±150 nm diameter were synthesized according to literature [35]. Zn(NO_3_)_2_.6H_2_O (5.875 g) and HMim (12.96 g) were dissolved in DMF (100 mL) separately. The HMim solution was then added to the zinc nitrate solution under vigorous stirring for 5 min, after which the mixture was heated in a Schott bottle at 140 °C for 4 h. The resulting white precipitate was separated by centrifugation (4000 rpm, 20 min) and washed 3 times with ethanol, followed by drying at 75 °C.

Stability of the ZIF-8 nanoparticles was tested in the fermented hydrolyzed wheat/hay broth. Initially, the suspended solids from the broth were removed by centrifugation (3500 rpm, 25 min) to obtain a clear brown supernatant. The pH of this supernatant was measured using a VWR (Haasrode, Belgium) pH1100L pH meter. ZIF-8 particles were dispersed in the supernatant for a certain time interval and recovered by centrifugation (3500 rpm, 25 min). The pH of the supernatant was then measured again, and XRD patterns of the ZIF-8 particles were recorded as described above (see Section 2.5). Chemical composition of the ZIF-8 powders was analyzed by Bruker Alfa FTIR equipped with an ATR crystal measuring from 4000 to 400 cm^−1^ with a resolution of 2 cm^−1^ and averaged over 64 scans.

## 3. Results

### 3.1. PV Performance in Fermented Wheat/Hay Straw Hydrolysate

**Figure 1:**
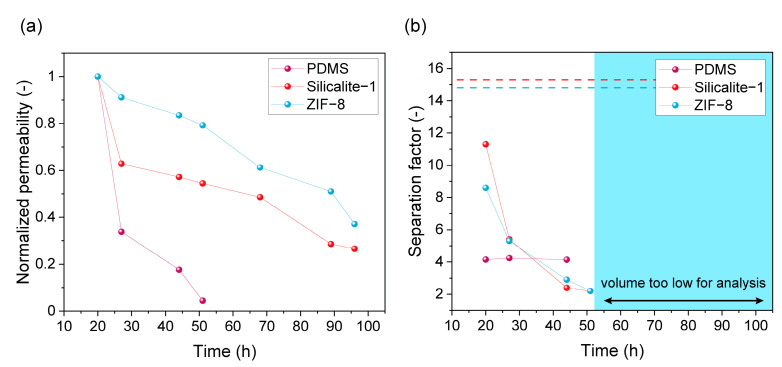
PV performance of the reference PDMS membrane as well as PDMS-based MMMs with silicalite-1 and ZIF-8 based fillers represented through (**a**) the normalized permeability and (**b**) the ethanol/water separation factor. After respectively 44 (PDMS) and 51 h (silicalite-1 and ZIF-8), the collected permeate volume was not sufficient anymore for HPLC-analysis and hence, no separation factor could be determined (region represented in blue). The separation factors of the ZIF-8 and silicalite-1 membrane for a synthetic pure ethanol–water mixture obtained from previous work are represented by a dashed line.

**Figure 2:**
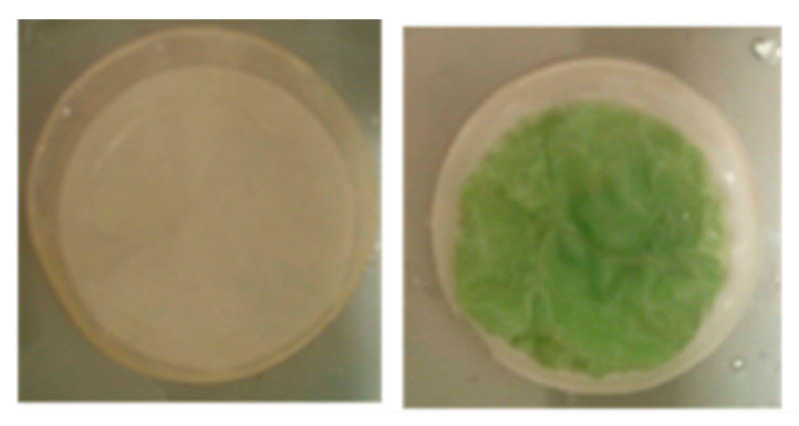
Visual appearance of the MMMs after PV of the fermented hydrolysate broth for (**left**) the silicalite-1 and (**right**) the ZIF-8 filled PDMS membrane.

PV experiments using the filled and unfilled PDMS membranes were performed with a fermented hydrolyzed wheat/hay straw broth. The normalized permeability and separation factor as function of time are summarized in Figure 1. It can be seen that the permeability decreased sharply for all membranes in the first hours, similar to the observations of Offeman et al. for ZSM-5 filled PDMS membranes [36]. After 51 and 100 h, practically no permeate was collected anymore from the unfilled and filled PDMS membranes, respectively. In addition, after a certain time, the amount of permeate collected was too low to run an HPLC analysis, making it impossible to determine the separation factor. Although the incorporation of the fillers (ZIF-8 or silicalite-1) initially resulted in a higher separation factor compared to the unfilled PDMS (attributed to the selective adsorption of ethanol in the pores of the hydrophobic filler materials), the initial separation factor for both MMMs in the real feed falls short of the value expected from the ideal separation factor obtained previously for a pure ethanol in water mixture (represented by the dashed lines). In addition, the separation factor of the MMMs shows a rapid decline to levels even below the reference unfilled membrane, which did not show any change in separation factor at all. Many phenomena could be at the origin of these effects, such as strong adsorption and subsequent pore blocking of certain broth components in the pores of the filler material or even degradation of the filler. While the silicalite-filled membrane kept its visual appearance after the PV run, the ZIF-8 filled membrane (Figure 2) showed signs of excessive swelling (wrinkled structure) and chemical changes (greenish color). This greenish color could be due to just adsorption of compounds in the ZIF-8 or due to a reaction of the Zn or HMim with a component from the fermentation broth.

From literature, it is clear that silicalite-1 is supposed to remain stable under these conditions [37]. The observed performance decline must thus be due to blocking of pores. Much less is known about MOFs under such conditions, and considering the concerns raised in literature on the stability of MOFs in certain conditions [38], the ZIF-8 MMM was studied in more detail.

**Figure 3:**
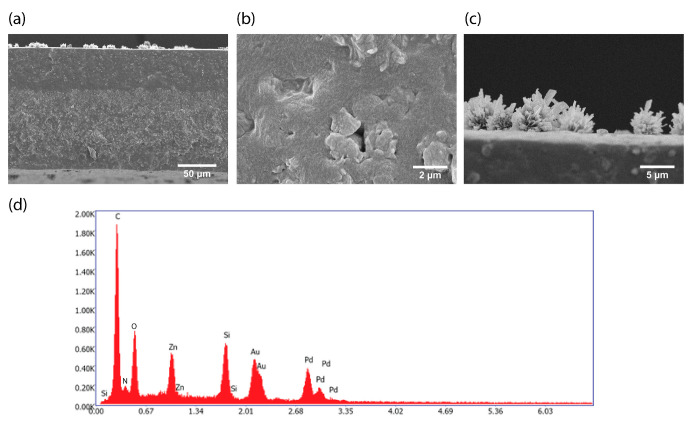
SEM cross-section analysis of the ZIF-8-filled PDMS membrane after PV with the fermented hydrolysate broth. (**a**–**c**) Cross-section images of (**a**) full cross-section, (**b**) detail image showing interfacial voids between the filler particles and the PDMS and (**c**) detail image of the surface fouling. (**d**) Representative EDX spectrum of the filler particles inside the cross-section. Identified elements are indicated on the spectrum with Au and Pd originating from the applied conductive coating.

SEM images of the ZIF-8 filled MMM after PV can be seen in Figure 3. The higher magnification image (Figure 3b) shows the presence of interfacial gaps between the polymer and the filler, which were not observed in the as-prepared MMMs [33]. The formation of such unselective interfacial voids suggests that the filler material was partly degraded during the PV. This could explain the observed drop in separation factor to below that of the pristine PDMS membrane. The chemical composition of the observed filler particles was analyzed via EDX-spectroscopy. The ratio of nitrogen to zinc (N/Zn), the two elements that are characteristic for ZIF-8 and which cannot be found in the PDMS matrix nor in the applied Au/Pd coating (to render the samples conductive), can be used as a characteristic to determine whether the original chemical structure of ZIF-8 is still present in the membrane. In a perfect ZIF-8 crystal, the N/Zn ratio should be 4, as each Zn^2+^ ion is tetrahedrally coordinated to 4 nitrogen atoms of 4 Mim molecules. After PV however, a N/Zn ratio of only 0.09 ± 0.09 is found (average over 4 point measurements on filler particles), suggesting that most of the HMim could have leached out as a consequence of filler degradation.

**Figure 4:**
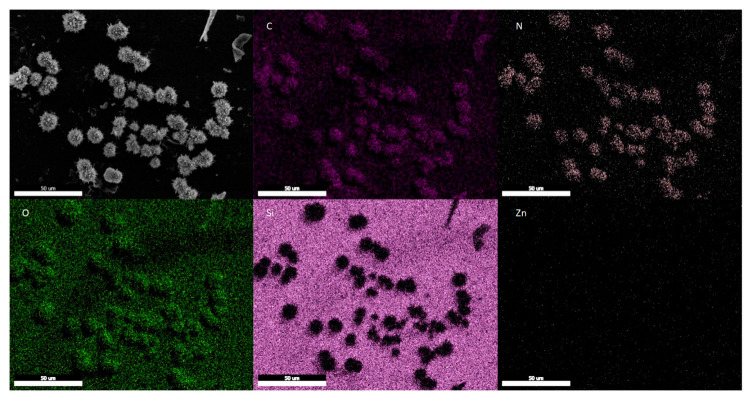
Topview SEM image of a ZIF-8 filled PDMS membrane after PV of the fermented hydrolysate broth as well as its SEM-EDX elemental mapping for C, N, O, Si and Zn (indicated on image).

Additionally, particulate fouling could be observed on the membrane surface (Figure 3c). A topview image of the membrane after PV (Figure 4) shows that many of these foulant particles are present on the surface of the membrane after PV. The SEM-EDX mapping shows that the foulant particles are mainly composed of carbon, nitrogen and oxygen. In addition, the overall EDX-spectrum of the mapped area shown in Figure 4 did not reveal the presence of any elements other than those associated to the polymer, filler or Au/Pd coating (Figure S3). Nevertheless, the exact origin of these particles is difficult to determine. Although their size could correspond to the size of yeast cells, their irregular shape suggests otherwise. Most probably, these particles are organic precipitates formed during the fermentation or PV process.

### 3.2. ZIF-8 Stability

#### 3.2.1. ZIF-8 Stability in the MMM

**Figure 5:**
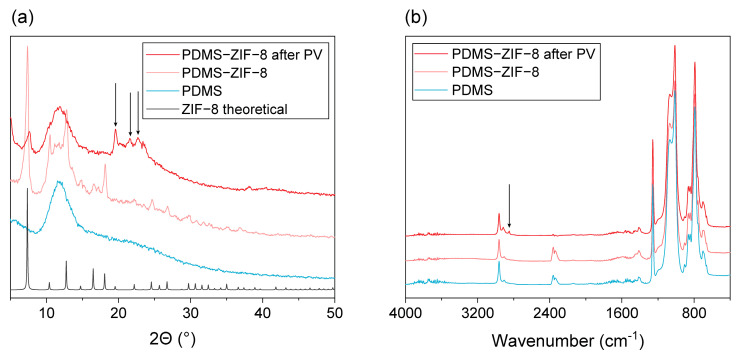
(**a**) XRD and (**b**) FTIR analysis of the PDMS and ZIF-8 filled PDMS membranes after PV of the fermented hydrolysate broth. Additional reflections/vibrations observed after PV are highlighted using arrows. Variations between the different spectra around 2344 cm^−1^ are caused by variations in CO_2_ concentration in the air during the measurements.

To investigate whether the crystalline structure of the ZIF-8 was compromised during the PV, XRD patterns of unfilled PDMS and ZIF-8 MMMs before and after PV were recorded (Figure 5a). The diffraction pattern of the ZIF-8 filled MMM shows strong reflections of ZIF-8, confirming its successful incorporation in the PDMS matrix, as expected [33]. After contact with the fermented hydrolysate feed for 120 h, most of these reflections either disappeared or reduced greatly in intensity. In addition, some other significant reflections were observed which cannot be attributed to ZIF-8. This suggests that the crystalline ZIF-8 shell of the ZIF-8 particles was completely degraded during the PV, which correlates with the SEM observations (Figure 3). It was not possible to attribute the new reflections to a 100% pure phase. This is probably due to multiple causes. First, the observed new reflections are relatively broad and masked by the strong background of the amorphous PDMS. Second, the fermented hydrolysate is a mixture containing many different compounds, making it highly possible that the newly formed material contains multiple phases and impurities.

Further membrane characterization was done via ATR-FTIR (Figure 5b). Apart from one very small peak at 2844 cm^−1^, all spectra are similar, showing only the vibrations of the PDMS matrix. Probably, the ATR-FTIR penetration depth is too limited to detect the filler in the bulk of the membrane. As the FTIR spectra show no difference, the green color thus must originate either from adsorption of feed components deep inside the membrane matrix or from a reaction with the building blocks of ZIF-8.

#### 3.2.2. ZIF-8 Nanoparticle Stability in the Fermented Hydrolysate Broth

As the PDMS matrix of the MMMs presented a barrier to study the influence of the interactions between the ZIF-8 and the fermentation broth, the influence of the fermented hydrolysate broth on the ZIF-8 nanoparticles was also studied ex-situ. The suspended solids from the fermented hydrolysate broth were removed by centrifugation to obtain a clear supernatant. The pH of this supernatant was found to be mildly acidic (pH 4.96). ZIF-8 particles were dispersed in this supernatant and recollected via centrifugation after a certain time to be analyzed via XRD and FTIR. The pH of the remaining supernatant was also measured again (see Table 1). As the breakdown of ZIF-8 would liberate 2-methylimidazolate, which can be protonated, a pH increase of the supernatant is an indicator for breakdown of the ZIF-8. As can be seen in Table 1, increasing the contact time between the ZIF-8 and the supernatant of the fermentation broth indeed leads to an increase in pH from 4.96 to 5.70, pointing towards a significant breakdown of ZIF-8 upon contact.

**Figure 6:**
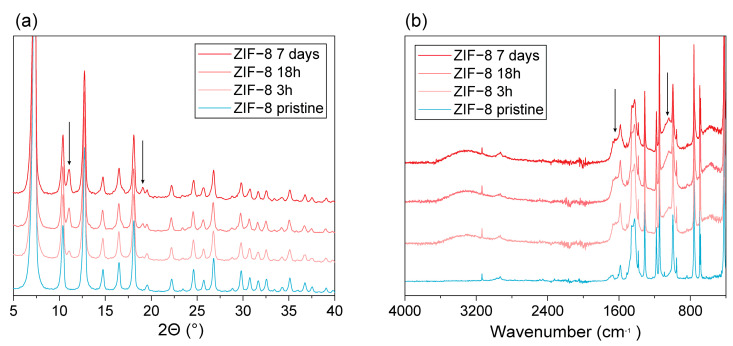
Characterization of ZIF-8 nanoparticles exposed for varying time to the fermented hydrolysate broth through (**a**) XRD and (**b**) FTIR. Arrows in the plot indicate major additional reflections or vibrations observed after broth exposure.

**Figure 7:**
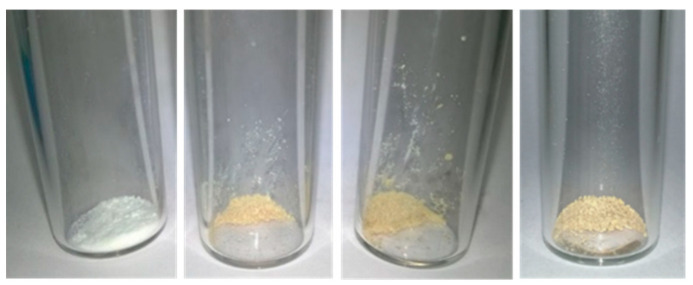
Visual appearance of the ZIF-8 powder after exposure for various lengths of time to the fermented hydrolysate broth, from left to right: pristine ZIF-8, 3 h, 18 h and 7 days exposure.

Since an unidentified phase was observed in the diffraction patterns of the ZIF-8filled MMMs after contact with the fermented hydrolysate, powder XRD of the reference ZIF-8 nanoparticles recovered from the fermented hydrolysate supernatant was also performed (Figure 6a). XRD analysis of these samples revealed two major things. (i) Two new reflections were detected at 11.1° and 19.1° (indicated using arrows). These reflections were already weakly visible after 3 h and became stronger with increasing contact time. Since major ZIF-8 reflections were preserved, it indicates that a new phase might have formed or that the ZIF-8 framework had undergone a partial transformation to another material. (ii) For a similar amount of analyzed sample, background intensity increased with increasing contact time, indicating the presence of an increasing amount of amorphous material, assumed to be due to partial degradation of the original material. This thus confirms that the increasing pH of the supernatant with ZIF-8 contact time results from framework destruction. Unfortunately, none of the two new reflections coincides with the unidentified reflections that were observed in the XRD of the ZIF-8 filled MMM. It is hypothesized that this could be due to the fact that conditions inside the MMM during PV are different from the fermented hydrolysate itself and that the two environments are thus not 100% equal. From the physicochemical properties of the PDMS (which are also reflected in the EtOH/H_2_O separation factor), it can be expected that the concentration of organics (EtOH, but also other more hydrophobic or volatile fermentation broth components) inside the membrane is much higher, which could have led to the precipitation of the material that caused the green color. The visual appearance of the ZIF-8 powder after contact and washing was also assessed (Figure 7). The white color of the virgin ZIF-8 powder turned to brown when brought into contact with the fermentation broth. This suggests that certain compounds from the fermentation broth adsorbed strongly within the ZIF-8 framework, which could also partially explain the higher background in the XRD patterns. The ZIF-8 powders were further analyzed via FTIR (Figure 6b). Aside from a broad hydrogen-bonded O-H stretching vibration around 3300 cm^−1^, two additional vibrations at 1650 and 1040 cm^−1^ were observed. While the former suggests some hydrophilic substance might be adsorbed onto or into the ZIF-8 compound, its exact composition in combination with the two latter vibrations is difficult to determine because of the broth complexity.

#### 3.2.3. General Remarks

The findings presented in this work raise multiple important issues in the development of novel membranes for PV. (i) Realistic feed streams are complex and can have many effects on the membrane and separation process that are often not well understood. Deeper understanding of these effects is crucial to improve the chances of novel membranes reaching the commercial stage. (ii) For the first time, it was demonstrated that MOFs, although very promising materials in theory, might have severe limitations in membrane separation when applied in realistic conditions. (iii) In literature, a multitude of approaches is used when studying realistic feed streams for PV, ranging from the addition of certain compounds to synthetic mixtures, to real fermentation broths. Also, within the latter, a lot of different approaches can be found, such as fixed cell bed fermentation broths, fermentation broths with ultrafiltration pre-treatment or pure fermentation broths without pre-treatment. Although each of these has their own advantages and disadvantages, this results in difficult comparison of data between different reports. However, this diversity in methods could also help to identify the solution to the observed performance reduction. Detailed understanding of the effects of realistic feed streams on novel membrane materials could provide the basis for the development of tailored feed pre-treatment to ensure long-term stable operation of novel high-performance membrane materials.

## 4. Conclusions

In comparison with unfilled PDMS membranes, (hollow) silicalite-1 and (mesoporous silica-coated) ZIF-8 filled PDMS offer considerably improved performance in the separation of (bio)ethanol from aqueous solutions via PV when using synthetic feeds. However, using a real fermentation broth as applied in this study, both unfilled and filled PDMS membranes show a big decrease in permeability, most probably due to the accumulation of feed components on the membrane surface and inside the polymer or filler pores. Importantly, the filled membranes also gradually lose their advantage in separation factor over the unfilled membrane. The case of the ZIF-8 membrane was further investigated in detail as the stability of MOFs in real PV conditions had so far been unexplored. It was shown that application of the ZIF-8 filled MMM in the broth resulted in a complete degradation of the crystalline structure of the MOF after 120 h of PV. Several additional reflections were found in XRD analysis, but it was not possible to assign them to a specific phase. Reference experiments testing the stability of ZIF-8 nanoparticles in the supernatant of the fermentation broth further confirmed that ZIF-8 is not stable in this acidic environment. Additionally, fouling was observed on the membrane surface via SEM. These foulants are thought to be of an organic nature as EDX analysis only showed the presence of carbon, nitrogen and oxygen. This research thus underlines the need to test newly developed membranes under realistic conditions to identify their full industrial potential.

## Figures and Tables

**Table 1:** pH of the fermented hydrolysate supernatant as a function of contact time with the ZIF-8 nanoparticles.

Contact Time (h)	pH
0	4.96
3	5.46
18	5.68
168	5.70

## Data Availability

Data is available upon request.

## Supplementary Materials

The following supporting information can be downloaded at: https://www.mdpi.com/article/10.3390/membranes13110863/s1, Figure S1: chemical structure of the RTV 615 PDMS kit; Figure S2: Schematic overview of the cross-flow PV setup; Figure S3: Overall EDX spectrum of the mapped area in Figure 4.
