# Supplementary material for: Stability of Filled PDMS Pervaporation Membranes in Bio-Ethanol Recovery from a Real Fermentation Broth
Source: Membranes (Basel). 2023 Oct 27;13(11):863. doi: 10.3390/membranes13110863 (PMC10673076; doi:10.3390/membranes13110863)
Supplement: Supplementary file 1 [file membranes-13-00863-s001.zip › membranes-2601074-supplementary.pdf]

membranes

Supporting information

Stability of Filled PDMS Pervaporation Membranes in Bio-Ethanol Recovery from a Real Fermentation Broth

Cédric Van Goethem [1], Parimal V. Naik [1], Miet Van de Velde [2], Jim Van Durme [3], Alex Verplaetse [2] and Ivo F. J. Vankelecom [1,*]

[1] Membrane Technology Group, Centre for Membrane Separations, Adsorption, Catalysis and Spectroscopy for Sustainable Solutions (cMACS), Department of Microbial and Molecular Systems, KU Leuven, Celestijnenlaan 200F, 3001 Leuven, Belgium
[2] Laboratory of Enzyme, Fermentation and Brewery Technology, Cluster for Bioengineering Technology, Department of Microbial and Molecular Systems, KU Leuven, Gebroeders De Smetstraat 1, 9000 Ghent, Belgium
[3] Research Group Molecular Odor Chemistry, KU Leuven Technology Campus Ghent, Gebroeders De Smetstraat 1, 9000 Ghent, Belgium
* Correspondence: ivo.vankelecom@kuleuven.be

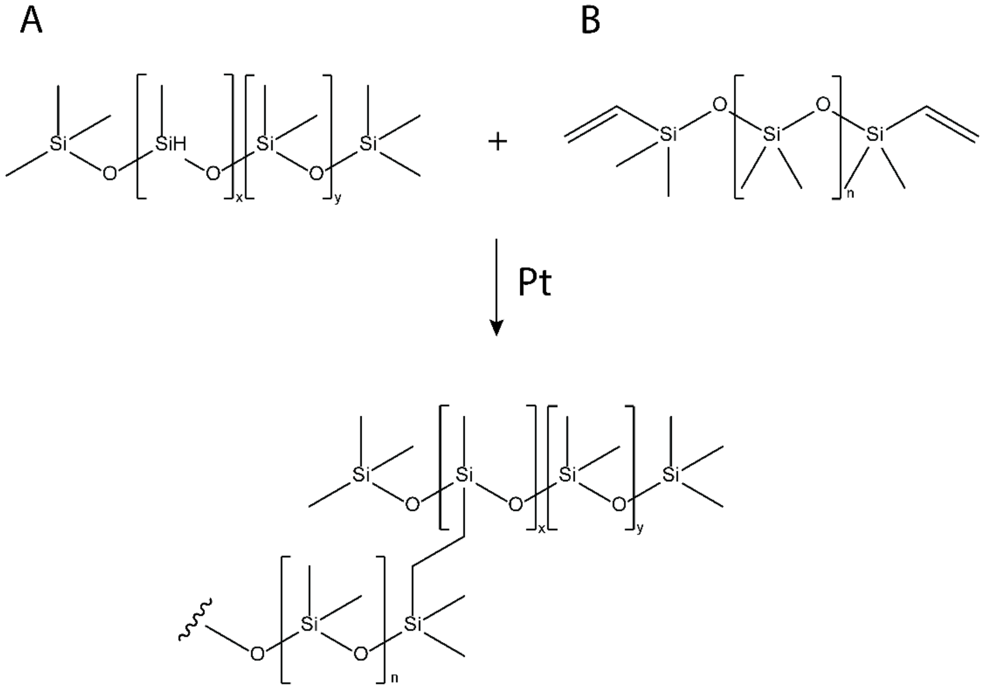

Figure S1. Presumed chemical structure of the two-component, Pt-catalyzed PDMS kit (RTV 615) used in this work, including the Si-H functional prepolymer (**A**) and vinyl terminated crosslinker (**B**).

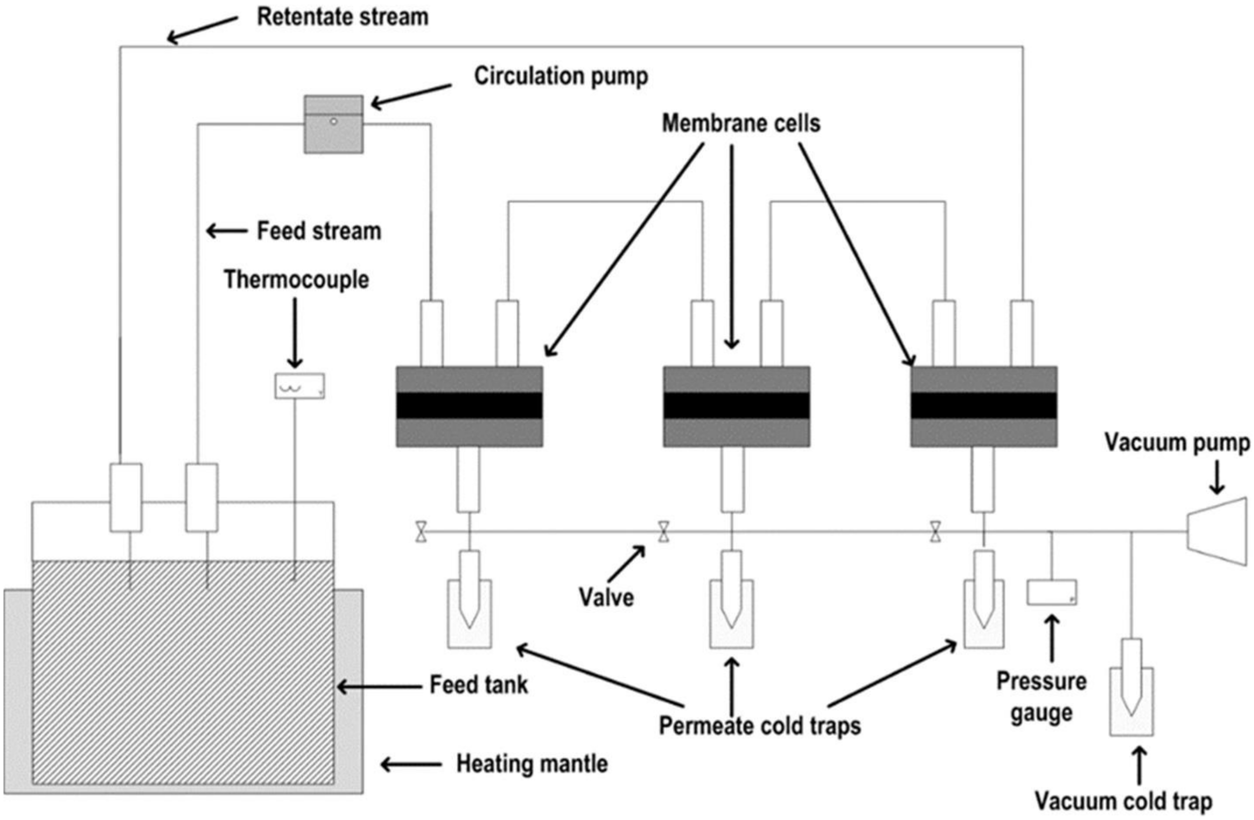

Figure S2. Schematic overview of the cross-flow pervaporation setup.

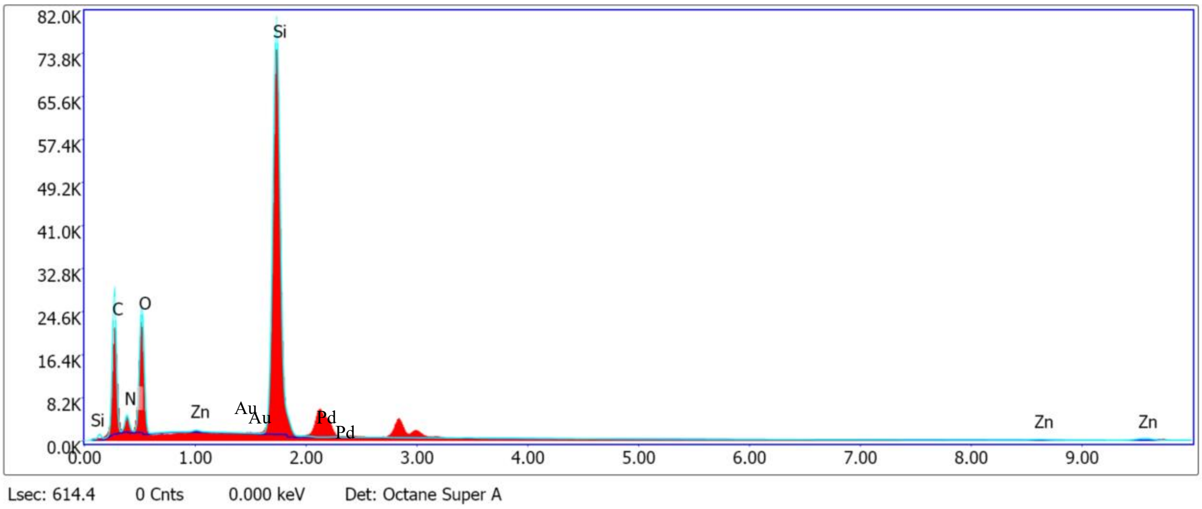

Figure S3. Overall EDX spectrum of the mapped area in Figure 4.
